# Hepatitis B At-Birth Dose Vaccine: An Urgent Call for Implementation in Ghana

**DOI:** 10.3390/vaccines6010015

**Published:** 2018-03-12

**Authors:** Yaw Asante Awuku, Mary Yeboah-Afihene

**Affiliations:** 1 Department of Medicine and Therapeutics, University of Cape Coast, Cape Coast, Ghana; 2 Department of Medicine, Kwame Nkrumah University of Science and Technology, Cape Coast, Ghana; mkyafihene.chs@knust.edu.gh

**Keywords:** hepatitis B infection, mother-to-child transmission, birth dose vaccine

## Abstract

Globally, approximately two billion people are infected with the Hepatitis B virus with attributable death estimated at about half a million people annually across the globe. Chronic hepatitis B infection is also an important public health problem in Ghana. The main mode of transmission in endemic regions is the perinatal route. Mother-to-child transmission can be reduced by antiviral therapy especially in the last trimester and adherence to the national immunization schedule. The World Health Organization recommends to add the birth dose vaccine to the current expanded program on immunization (EPI) in all countries but especially for endemic regions. The evidence for the efficacy of the birth dose HBV vaccine is overwhelming and there is an urgent need for its introduction into the current EPI schedule in Ghana.

## 1. Introduction

The discovery of hepatitis B virus (HBV) by the Nobel Prize winner Baruch S. Blumberg has contributed significantly to the development of viral diagnostics and vaccines [1]. One of the global health problems in this era is chronic hepatitis B infection. It is estimated that 257 million people are chronically hepatitis B surface antigen (HBsAg) positive [2]. Although a vaccine preventable disease, the burden of chronic hepatitis B infection is showing an increasing trend across the world especially in sub-Saharan Africa [3]. The annual HBV attributable death is estimated at about half a million people across the globe [4]. The estimated chronically infected HBV population in Western Europe and North America is less than 1%. The overall lifetime risk of HBV infection on the continents of Africa and Asia are very high [5]. The natural history of chronic HBV infection suggests that about 15–25% infected individuals may develop cirrhosis, liver failure, and hepatocellular carcinoma. As a vaccine preventable disease from current available evidence, there is an urgent need to fully implement the World Health Organization HBV vaccination protocol including the at-birth dose vaccine to reduce the risk of perinatal transmission in Ghana.

## 2. Prevalence of Chronic Hepatitis B Infection

The estimated prevalence of hepatitis B infection is high (equal or more than 8% prevalence of hepatitis B surface antigen in the population) in sub-Saharan Africa [6]. This public health problem requires urgent attention across the globe but especially in Ghana [7,8]. HBV in endemic regions is transmitted mostly perinatally (mother-to-child) and horizontally during early childhood. It is also known that for hepatitis B e-antigen positive pregnant women perinatal transmission is high but this is unlikely to be a big problem as majority of the patients in our sub region are e-antigen negative. The age of acquisition of HBV infection is an important predictor for developing a chronic disease. The risk decreases from 80–90% for perinatal infection to 30–60% in early childhood, 5–10% for ages 5–20 years, and 1–5% for adults older than 20 years [9]. In Ghana, the sero-prevalence of hepatitis B infection varies according to the study population and the region where the study was conducted. It ranges from as low as 3.5% among sickle cell patients in the Greater Accra region to as high as 20.9% in a community in the Ashanti region [10]. A systematic review involving studies published in peer review journals from 1995–2015 estimated the average prevalence as 12.3% with majority of the studies involving blood donors [10]. The natural history of HBV infection is progression to cirrhosis, liver failure, and—in a minority if patients—to hepatocellular carcinoma. The high prevalence in Ghana if not addressed will lead to high HBV related morbidity and mortality with associated high cost of health care. There is currently routine testing for HBV at the ante-natal clinics in most African countries, including Ghana, but there is no government policy on at-birth dose vaccine [8,9] which has demonstrated significant reduction in perinatal transmission of HBV in other jurisdictions [9,10].

## 3. Hepatitis B Infection as a Vaccine Preventable Carcinogen

Hepatocellular carcinoma (HCC) incidence is higher in Africa compared to the rest of the world because of the higher prevalence of chronic viral hepatitis. HCC is also an important cause of cancer related death in Africa and South East Asia [11]. HCC incidence globally in 2015 was 854,000 and 810,000 deaths contributing to 20,578,000 disability adjusted life year (DALYs). Globally, HBV accounted for 265,000 liver cancer deaths (33%), alcohol for 245,000 (30%), and Hepatitis C virus (HCV) for 167,000 (21%) [12]. Chronic HBV infection is the most common global cause of HCC and the predominant etiology in China and Africa [13]. In Ghana HCC is considered the fourth most important cancer according to the Komfo Anokye Teaching Hospital registry and ninth most important cancer in males from the cancer registry at Korlebu Teaching Hospital [14,15]. The biological basis of HBV as an etiologic agent has been well described. The proteins of HBV and integration of HBV DNA into the genome of the host has been implicated in the carcinogenesis process. These molecules are said to have a potent carcinogenic effect by driving the cis and trans oncogenic signals in the host liver cells [13]. It has also been shown that HCC tends to occur at an earlier age in Africans compared to other settings. This early age HCC onset was demonstrated to have a strong association with chronic hepatitis B infection [16]. We have recently shown that in general, care and outcome of HCC in Africa is poor, with the exception of Egypt where there are policies and structures in place to improve care for patients with HCC [17].

There is the need to focus on prevention through education and a special vaccination program for all in Ghana.

## 4. Prevention of Hepatitis B Mother-to-Child Transmission

HBV infection can be transmitted from mother-to-child during the perinatal period. Pregnancy and early childhood are the periods where acquisition of HBV has a high risk of becoming a chronic disease [9]. The natural history of HBV transmission is that about 90% of infants from hepatitis B e antigen (HBeAg) positive mothers will be infected whilst 5–20% of those from HBeAg negative mothers will be infected [18]. Maternal HBV DNA level is the single most important predictor of mother-to-child transmission (MTCT) and levels of 106 to 108 copies/mL are associated with very high transmission risk. MTCT occurs during pregnancy, especially in third trimester and intrapartum period. Therefore, efforts to use antivirals to reduce viral load and at-birth vaccination within 24 h of the newborn combined with the current EPI protocol in Ghana will reduce transmission significantly [18,19].

## 5. Immunization Schedule and the Need for HBV Birth Dose Vaccine in Ghana

Immunization is globally utilized as a means of preventing infections especially among children. Universal infant immunization for HBV was recommended following the overwhelming success of this approach in Taiwan [20]. The World Health Organization (WHO) unequivocally recommended its incorporation into the expanded program on immunization (EPI) for all countries, especially in Africa in 1991 [21]. Ghana had a delayed response to this call and only adopted HBV vaccination in children as part of the EPI in 2002 to reduce the HBV menace. The evidence for the prevention of perinatal transmission of HBV by the birth dose vaccine is overwhelming as demonstrated by the Chinese government through a public private partnership with the Global Alliance for Vaccines and immunization (GAVI) [22].

The EPI schedule for Ghana recommends Bacille Calmette–Guerin (BCG) at-birth; oral polio vaccine (OPV) at birth, 6, 10, and 14 weeks of age (OPV-0, OPV-1, OPV-2, and OPV-3 respectively); three doses of diphtheria, pertussis, tetanus, *Haemophilus influenzae* type B, and hepatitis B (DPT/HiB/HepB) 5 in 1 vaccine at 6, 10, and 14 weeks of age. Measles and yellow fever vaccines are given at nine months of age [23].

Examining the current schedule shows that there are already some vaccines given at birth and therefore introduction of the birth dose vaccine for HBV will not disrupt the EPI program in Ghana. The hepatitis B monovalent vaccine can be given together with OPV-0 and BCG. However, this needs to be given ideally within 24 h of the baby’s delivery.

This modification to the immunization schedule in terms of timing is not problematic as it only introduces another at-birth dose vaccine at an extra cost. The health sector should be pro-active in leading the government towards the introduction of the birth dose vaccine for HBV as it has been proven efficacious in the prevention of mother-to-child transmission in many other countries [24,25,26]. There is the need to establish a public private partnership with GAVI and government of Ghana to make the implementation of the birth dose vaccine a reality as soon as possible. We also do recommend the need to explore other local and international funding to make this vaccine introduction sustainable once the initiative takes off.

## 6. Conclusions

There is an urgent need for the introduction of the at-birth dose vaccine for HBV in our current EPI schedule to help fully fight the menace of HBV infection in Ghana. In parallel with at-birth vaccination, increased surveillance programs should be implemented to facilitate accurate monitoring of HBV sero-prevalence in different regions of Ghana.
